# Four-point dermo-fascial fixation for umbilical reconstruction following keloid excision: clinical experience in 16 patients

**DOI:** 10.3389/fsurg.2026.1904266

**Published:** 2026-08-19

**Authors:** Peiyu Li, Haitao Xiao, Xuewen Xu, Ru Wang

**Affiliations:** 1 Department of Burn and Plastic Surgery, West China Hospital, Sichuan University, Chengdu, Sichuan Province, China; 2 West China School of Medicine, Sichuan University, Chengdu, Sichuan Province, China

**Keywords:** keloid excision, laparoscopic port-site scar, umbilical keloid, umbilical reconstruction, umbilicoplasty

## Abstract

**Background:**

Umbilical keloids after transumbilical laparoscopic surgery are difficult to manage because excision may leave a contour defect in a concave, mobile, and scar-prone region. Reconstruction must restore a recessed umbilical contour while minimizing additional incisions and scar burden.

**Methods:**

This brief report describes a four-point dermo-fascial fixation technique in 16 patients with round, bulging, or transversely elongated umbilical keloids after laparoscopic surgery. In all patients, the keloid margin extended no more than 1.0 cm beyond the visible umbilical rim. After limited circumferential defatting, adjacent umbilical tissue was anchored to the residual fibrous umbilical stalk and linea alba using a four-point configuration. All patients received postoperative radiotherapy and topical scar therapy.

**Results:**

The patients were 35–72 years old, and photographic follow-up ranged from 6 to 15 months. All patients maintained a recessed umbilical contour during the available follow-up. No wound complications were observed, and no clinically evident keloid recurrence occurred after combined surgical reconstruction, postoperative radiotherapy, and topical scar therapy. A transverse scar or crease remained visible in some cases.

**Conclusion:**

The four-point dermo-fascial fixation technique may provide a simple local-tissue option for scar-control-oriented umbilical reconstruction after keloid excision, although it should not be considered an ideal aesthetic umbilicoplasty. Its main value lies in its simplicity, use of adjacent local tissue, avoidance of graft harvest or complex flap design, and potential reproducibility when scar control and technical practicality are prioritized. Larger studies with longer follow-up and standardized scar assessment are required to clarify long-term recurrence and aesthetic outcomes.

## Introduction

The umbilicus is a central landmark of the abdomen, formed from the healed umbilical cord (1), and a common access site for laparoscopic surgery (2). Although transumbilical incisions are generally intended to minimize visible scarring, keloid formation at this site can obliterate the normal umbilical depression and cause pruritus, pain, hygiene difficulty, and cosmetic concern. Management is challenging because the umbilicus is a concave and mobile region where moisture retention, epithelial debris, chronic inflammation, and wound tension may contribute to persistent symptoms and recurrence.

Umbilical reconstruction after keloid excision is therefore different from routine cosmetic umbilicoplasty. In these patients, the reconstructive goal is not only to recreate an acceptable umbilical depression, but also to minimize additional incisions, wound tension, and scar burden. Published approaches to umbilical reconstruction after keloid excision include full-thickness skin grafting, local transposition flaps (3), and flap-based fixation to the linea alba (4). Keloid-specific series have reported satisfactory restoration of umbilical depth and relatively low recurrence rates. However, interpretation is limited by small sample sizes, heterogeneous follow-up, and the frequent use of postoperative radiotherapy, steroid therapy, or other scar-management measures. Full-thickness skin grafting can provide coverage when primary closure is difficult, but introduces a donor site and potential graft-related complications. Local or triangular flap techniques provide vascularized tissue and may improve scar concealment and three-dimensional contour, but require additional incisions, tissue rearrangement, and more complex wound geometry.

Evidence from routine aesthetic and reconstructive umbilicoplasty further demonstrates that limited periumbilical defatting and fixation of the umbilical dermis or stalk to the rectus fascia are established reconstructive principles. In a systematic review, periumbilical defatting was described in 8 of 10 techniques, whereas dermis-to-fascia fixation was used in 4 of 10 techniques (5). Comparative studies have also shown that incision geometry influences umbilical shape, depth, natural appearance, and scar visibility, with discontinuous U- or V-based designs potentially reducing the risk of a conspicuous cicatricial ring associated with uninterrupted circular or oval incisions. However, these studies were conducted mainly in patients undergoing abdominoplasty or abdominal-flap breast reconstruction and may not be directly generalizable to patients predisposed to keloid formation.

Against this background, the purpose of the present report is not to claim novelty for periumbilical defatting or dermal fixation to the umbilical base itself. Rather, we describe a defined four-point dermo-fascial anchoring configuration for selected post-laparoscopic umbilical keloids. The technique uses adjacent local tissue and a transverse linear closure without graft harvest or flap transposition. We also summarize the early clinical outcomes of 16 patients treated with this reconstructive approach and standardized postoperative scar management.

## Method

### Clinical setting and patient selection

This retrospective descriptive series included 16 patients who underwent complete excision of a post-laparoscopic umbilical keloid followed by immediate reconstruction using four-point dermo-fascial fixation. No formal morphologic classification system was used. The technique was selected for limited umbilical keloids that could be completely excised while preserving sufficient adjacent umbilical tissue for tension-minimized transverse primary closure.

On retrospective review of the clinical records and preoperative photographs, all treated lesions had a round, bulging, or transversely predominant configuration, and the visible keloid margin extended no more than 1.0 cm beyond the umbilical rim. The technique was not selected for lesions extending more than 1.0 cm beyond the umbilical rim, vertically predominant lesions extending outside the umbilical unit.

Clinical information, including patient demographics, the indication for and type of the previous laparoscopic procedure, keloid dimensions, lesion duration, previous treatments, and postoperative follow-up, was retrospectively reviewed. Written informed consent was obtained from all patients for treatment, clinical photography, and publication of de-identified clinical images.

### Surgical technique

Procedures were performed under local anesthesia with sedation for smaller lesions or under general anesthesia for larger lesions. Patients were placed in the supine position. Standard sterile preparation was performed, and prophylactic antibiotics were administered.

**Figure 1:**
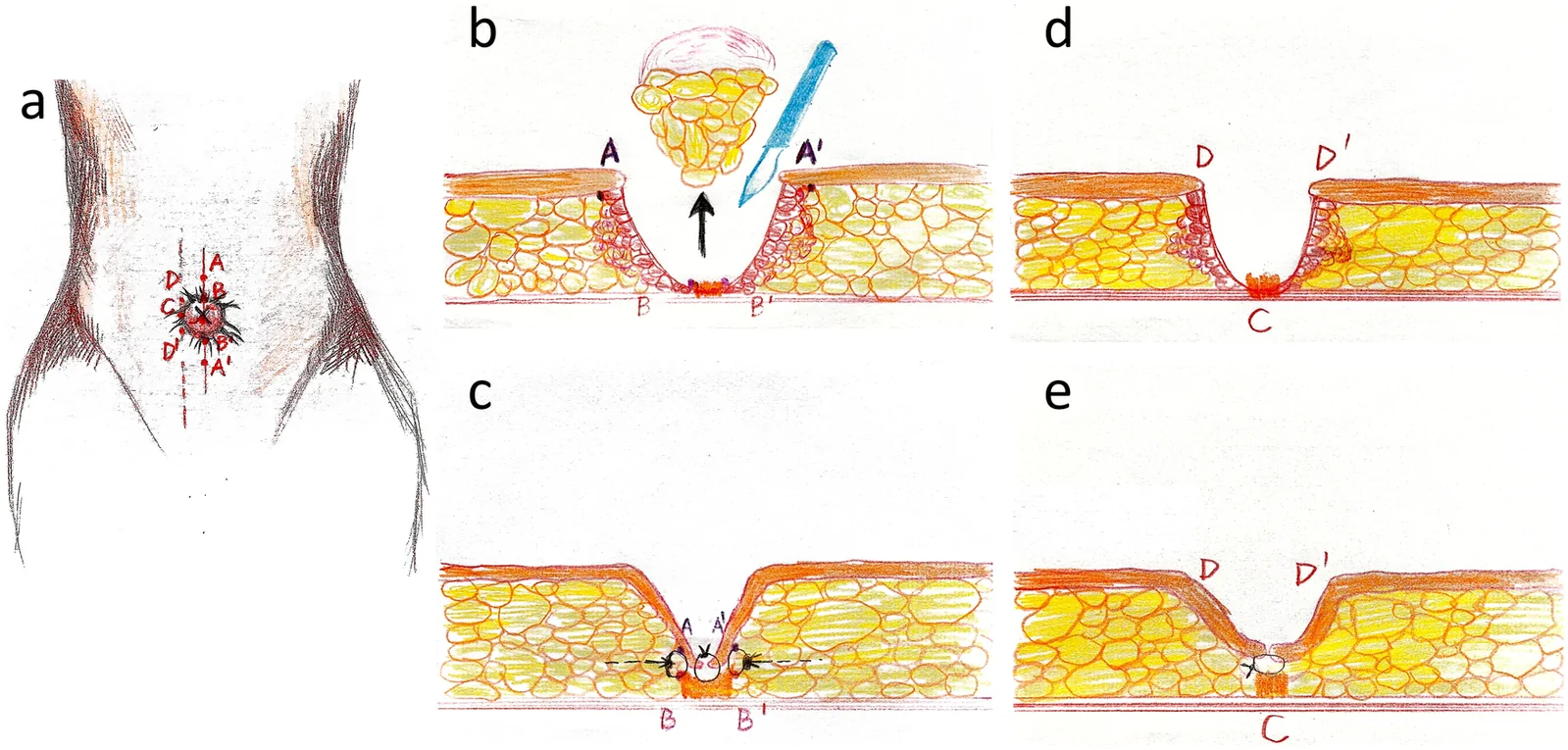
Schematic illustration of the four-point dermo-fascial anchoring technique for umbilical keloid excision and reconstruction. **(a)** Preoperative marking showing the umbilical keloid and planned transverse or elliptical excision. Points A, A’, B, B’, C, D, and D’ indicate anchoring and closure landmarks. **(b)** En bloc excision of the umbilical keloid. Subcutaneous fat is selectively resected within a 0.5–1.0 cm circumferential zone beyond the keloid margin to facilitate mobilization of the adjacent umbilical skin and formation of a recessed contour. B and B’ denote the superior and inferior borders of the deep fascial umbilical stalk. **(c)** Dermo-fascial anchoring. Two 3-0 PDS sutures approximate A-B and A’-B’, forming the primary tension-bearing line. An additional 3-0 PDS suture at C-D-D’ reinforces the depth and symmetry of the reconstructed umbilicus. A mirror-image 3-0 PDS anchoring suture is placed on the 3-o’clock side using the same principle. Together, these lateral anchoring sutures complete the four-point anchoring configuration. The deep and superficial fascial layers at the central point of the umbilicus are sutured using 5-0 PDS. **(d)** Preparation of the anchoring base. The deep fascial umbilical stalk and underlying linea alba are exposed, and the surgical bed is contoured for stable depression formation. **(e)** Final configuration after closure. Layered tension-reduction suturing and skin closure result in a transverse linear scar positioned at the base of the reconstructed umbilical depression.

1. Preoperative marking and keloid excision (Figure 1a):

With the patient in the supine position, the keloid margin was marked and a transverse or elliptical excision was designed to allow horizontal closure. The keloid was excised *en bloc* down to the subcutaneous layer. After complete removal of the lesion, subcutaneous fat was selectively resected within a circumferential zone approximately 0.5–1.0 cm beyond the keloid margin. This limited defatting allowed mobilization of the adjacent umbilical skin and helped create sufficient space for a stable postoperative depression.

2. Four-point dermo-fascial anchoring (Figures 1b,c):

The residual fibrous umbilical stalk and underlying linea alba were exposed as the anchoring base. In the schematic illustration, points B and B' correspond to the superior and inferior borders of the deep fascial umbilical stalk. Points A and A' were located on the superficial fascial layer at the outer margin of the mobilized area, and were paired with B and B', respectively. Two 3-0 polydioxanone (PDS) sutures were placed to anchor B to A and B' to A', creating the primary tension-bearing horizontal line and defining the depth of the reconstructed umbilicus. An additional 3-0 PDS anchoring suture was then placed at C-D-D', where D and D' represent the midpoints between the central point of umbilical stalk and the 9-o'clock margin. A mirror-image 3-0 PDS anchoring suture was placed on the 3-o'clock side using the same principle. Together, these lateral anchoring sutures reinforced symmetric dermal fixation to the deep fascia and complete the four-point anchoring configuration. The deep and superficial fascial layers at the central point of the umbilicus were sutured using 5-0 PDS.

3. Wound closure and postoperative care:

After the umbilical depression had been established, layered tension-reduction suturing was performed. The skin was closed with simple interrupted or running intradermal sutures, leaving a transverse linear scar at the base of the reconstructed umbilical depression (Figures 1d,e). Postoperative adjuvant scar management was used in all patients. Early postoperative radiotherapy was initiated on postoperative day 1 with a total dose of 20 Gy according to our institutional protocol, followed by topical anti-scar therapy. The radiotherapy plan was delivered with attention to limiting exposure of surrounding abdominal structures.

### Follow-up and descriptive assessment

Patients were followed by clinical examination and photographic documentation. Postoperative findings summarized in this report included maintenance of a central recessed umbilical contour, wound dehiscence, infection, skin necrosis, need for revision surgery, visible transverse scar or crease, contour irregularity, and keloid recurrence during the available follow-up period.

Keloid recurrence was defined as progressive raised scar tissue extending beyond the original excision margin. Hypertrophic scarring confined to the incision was recorded separately and was not classified as keloid recurrence. Photographic follow-up was used to document the postoperative umbilical contour and scar appearance.

### Statistical analysis

Because this was a small descriptive clinical report of a surgical technique, no inferential statistical analysis was performed. Continuous variables are presented descriptively, and categorical findings are summarized as counts.

## Results

**Figure 2:**
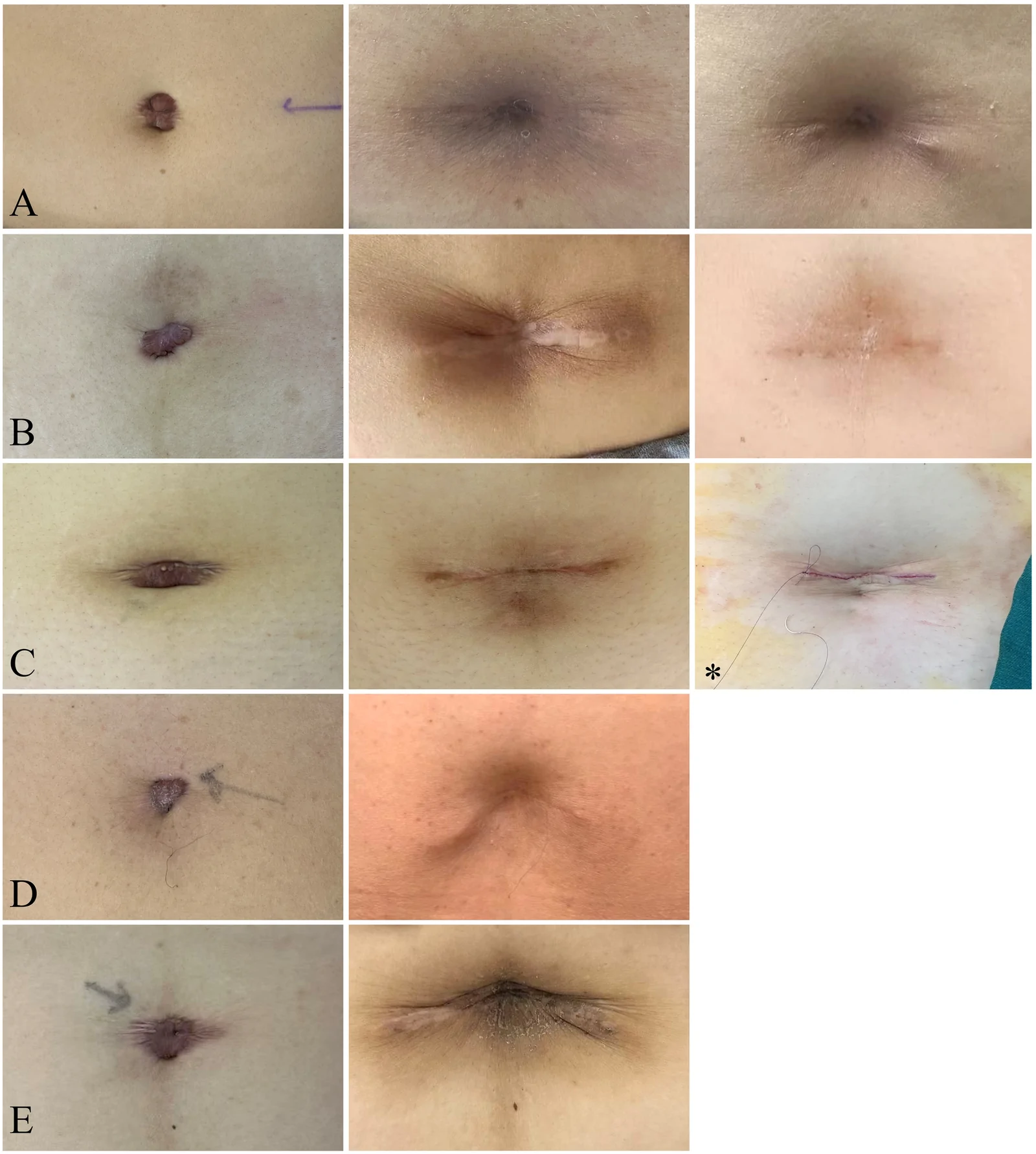
Preoperative (left column), 6-month postoperative (middle column), and 15-month postoperative (right column) photographs of five patients **(A–E)** who underwent the described umbilicoplasty technique. Panel * shows the immediate postoperative view of the umbilical keloid​ in a 35-year-old female patient (resulting from a prior laparoscopic teratoma surgery).

The four-point dermo-fascial fixation technique was applied in 16 patients aged 35–72 years, with BMI values ranging from 20.54 to 31.78 kg/m². The duration of the umbilical keloids ranged from 1 to 7 years. All lesions met the practical anatomical selection criteria described above: the keloid margin extended no more than 1.0 cm beyond the visible umbilical rim, sufficient adjacent tissue remained for tension-minimized transverse primary closure, and no patient required flap or graft reconstruction. On retrospective review, the estimated horizontal span of the umbilical unit was no longer than 4 cm. The previous laparoscopic procedures included laparoscopic cholecystectomy in 12 patients, laparoscopic ovarian surgery in 3 patients, and laparoscopic surgery for uterine leiomyoma in 1 patient. Histopathological examination confirmed keloid in all 16 excised specimens. Concomitant epidermal cysts were identified in four patients (4/16, 25%). No specimen was diagnosed as an isolated epidermal cyst or another lesion without keloid formation. Photographic follow-up ranged from 6 to 15 months (Figure 2). The final appearance was characterized by a transverse crease or linear scar at the base of the reconstructed umbilical depression, with mild lateral contour irregularity in some patients. There were no cases of conspicuous periumbilical ring scars or distortion. No wound dehiscence, infection, or skin necrosis occurred, and no patient required revision surgery. No keloid recurrence or hypertrophic scar was observed during the available follow-up. Individual patient characteristics, lesion dimensions, duration, previous treatments, and descriptive postoperative findings are summarized in Table 1.

**Table 1:** Clinical characteristics, pathological findings, postoperative scar management, and outcomes of the 16 patients.

Variable	Value
Number of patients	16
Age range, years	35–72
Female sex	16/16
BMI, kg/m²	20.54–31.78
Duration of keloid, years	1–7
Maximum transverse lesion diameter, cm	< 4
Previous treatment	3 patients received steroid injection
Laparoscopic cholecystectomy	12/16
Laparoscopic ovarian surgery	3/16
Laparoscopic myomectomy	1/16
Histologically confirmed keloid	16/16
Concomitant epidermal cyst	4/16
Postoperative radiotherapy	16/16; initiated on POD 1, total 20 Gy
Follow-up	6–15 months
Topical scar therapy	Yes
Wound dehiscence	0/16
Infection	0/16
Skin necrosis	0/16
Revision surgery	0/16
Keloid or hypertrophic scar recurrence	0/16

BMI, body mass index; POD, postoperative day.

## Discussion

In this brief clinical report, four-point dermo-fascial fixation was used for umbilical reconstruction after keloid excision in 16 patients with post-laparoscopic umbilical keloids (6). During 6–15 months of photographic follow-up, all patients maintained a central recessed umbilical contour, and no wound dehiscence, infection, skin necrosis, revision surgery, or clinically evident keloid recurrence was observed. These early findings support the feasibility of this local-tissue reconstructive approach in selected patients, although its independent effect on recurrence cannot be determined because all patients received postoperative adjuvant therapy.

Histopathological examination confirmed keloid in all specimens, while concomitant epidermal cysts were identified in four patients. Epidermal cysts may promote recurrent local irritation or inflammation within the moist and occluded umbilical environment and may therefore contribute to the development or persistence of keloid formation. Although we suspect an association, the present retrospective series cannot establish a causal relationship between the associated cysts and keloid development. These findings also emphasize the importance of complete excision and routine histopathological examination of umbilical keloid specimens.

The management of umbilical keloids is challenging because the umbilicus is a concave and mobile region. Retention of epithelial debris, chronic inflammation, infection, and abdominal wall motion may contribute to fibroproliferation and recurrence (7). Therefore, complete excision, control of local inflammation, tension-minimized closure, and postoperative scar management are important components of treatment.

Against this background, the main technical challenge after umbilical keloid excision is that the surgeon must restore a concave umbilical contour in a region with limited local tissue and a high tendency toward abnormal scarring. Simple excision may remove the keloid but can flatten or distort the umbilicus. In the present technique, limited circumferential defatting creates space for depression formation, while dermo-fascial fixation to the linea alba and residual fibrous umbilical stalk provides a stable anchoring base. The lateral fixation sutures reinforce symmetry and help maintain the recessed contour. Because the final closure is transverse and linear, the wound is relatively easy to approximate and postoperative scar care is straightforward.

Previously reported approaches to umbilical reconstruction after keloid excision demonstrate different clinical trade-offs. Tsujimoto et al. (2) reported four patients treated with full-thickness skin grafting and postoperative irradiation, with no recurrence during 6 months of follow-up; however, this approach introduced a donor site and the possibility of graft-related complications. Kwon et al. (3) described transposition-flap reconstruction in 10 patients and reported no recurrent keloid nodules during a mean follow-up of 10.9 months, although four patients required postoperative triamcinolone treatment for mild hypertrophic change or pruritus. Dohi et al. (8) reported 34 umbilical keloids treated using excision, umbilicoplasty when required, postoperative radiotherapy, and scar self-management, with three recurrences during 24 months of follow-up. These series suggest that satisfactory contour restoration and recurrence control can be achieved using different reconstructive strategies. Nevertheless, direct comparison is difficult because lesion severity, reconstructive indications, adjuvant treatment, follow-up duration, and definitions of recurrence differed among studies. However, these techniques may require graft harvest, broader undermining, additional incisions, or more complex flap geometry. Although scar length and scar concealment are major aesthetic concerns for both surgeons and patients, in keloid-prone patients recurrence prevention and controllable wound healing are often equally important. U-shaped or multiple-flap designs may hide scars more effectively in standard umbilicoplasty, but curved incisions and flap corners can create suture-line angles where hypertrophic change may be harder to control. Avoiding a continuous circular incision may reduce the risk of a conspicuous contracted ring scar (9). More recently, Kandulu described neo-umbilicus creation during abdominoplasty using selective defatting and dermo-fascial fixation, with adjunctive micro-fat grafting in selected patients to refine umbilical depth and three-dimensional contour. Although this approach provides useful contemporary context, it was developed for aesthetic abdominoplasty rather than for keloid-prone wounds and may therefore not be directly generalizable to reconstruction after keloid excision (10). The value of the present technique is therefore not that it replaces established reconstructive options, but that it provides a simple, reproducible, scar-control-oriented alternative for appropriately selected lesions. The present series does not demonstrate superiority over graft- or flap-based reconstruction, and those methods may remain preferable when the defect is too large for tension-minimized primary closure.

Patient selection is important because the feasibility of this technique depends on the amount and mobility of the remaining adjacent umbilical tissue. In the present series, the keloid margin extended no more than 1.0 cm beyond the visible umbilical rim, and the estimated horizontal span of the umbilical unit was no longer than 4 cm. These anatomical conditions allowed complete excision followed by transverse primary closure without excessive tension.

The technique should not be extrapolated to all umbilical keloids. Lesions extending more than 1.0 cm beyond the umbilical rim, vertically predominant lesions extending outside the umbilical unit, circumferential lesions, or defects with insufficient residual tissue may not permit tension-minimized primary closure and may be better managed using a local flap or skin graft. In practice, the amount and mobility of the remaining tissue and the anticipated closure tension are more important than lesion diameter alone. The 1-cm threshold used in this series should be regarded as an experience-based practical criterion rather than a validated universal cutoff.

The absence of recurrence in this series should be interpreted cautiously. Adjuvant therapy is particularly relevant in keloid surgery. Postoperative radiotherapy has been reported to reduce recurrence after keloid excision, including in umbilical lesions (8, 11). Recent meta-analytic evidence also supports surgical excision followed by adjuvant radiotherapy as an effective combined treatment strategy for keloids (12). In our series, all patients received postoperative radiotherapy beginning on postoperative day 1, with a total dose of 20 Gy, followed by topical scar therapy. Accordingly, the absence of recurrence in this small series should be interpreted as the outcome of combined treatment rather than the effect of the surgical technique alone.

This technique also has aesthetic limitations. Studies of normal and reconstructed umbilical morphology generally identify a small oval or vertically oriented depression, often with superior hooding, as aesthetically preferable. By contrast, the present technique may leave a visible transverse crease or linear scar at the base of the reconstructed umbilicus. In the upright position, this scar configuration can simulate the superior hooding that characterizes an aesthetically favorable umbilicus. However, mild lateral contour asymmetry may also occur, though this can be corrected with subsequent touch-up procedures. It should therefore be regarded as a scar-control-oriented reconstructive option rather than an ideal cosmetic umbilicoplasty (5, 13, 14).

In conclusion, four-point dermo-fascial fixation may offer a simple and reproducible local-tissue approach for scar-control-oriented umbilical reconstruction after keloid excision. The technique is not intended as an ideal aesthetic umbilicoplasty and may not be suitable for extensive or vertically elongated lesions. Because all patients received adjuvant radiotherapy and topical scar therapy, this series does not establish an independent anti-recurrence effect of the reconstructive technique. Larger studies with longer follow-up, standardized scar and aesthetic assessments, and comparison with established reconstructive methods are needed to clarify its long-term durability, recurrence profile, and patient-perceived outcomes.

## Data Availability

The original contributions presented in the study are included in the article/Supplementary Material, further inquiries can be directed to the corresponding author.
